# Supplementary material for: Complexome profiling of the Chlamydomonas psb28 mutant reveals TEF5 as an early PSII assembly factor
Source: Plant Cell. 2025 Mar 17;37(6):koaf055. doi: 10.1093/plcell/koaf055 (PMC12164590; doi:10.1093/plcell/koaf055)
Supplement: koaf055_Supplementary_Data [file koaf055_supplementary_data.zip › psb28_interactive.zip!/plots/intensity/qualityPlots/peptides/ALB3.2_Cre17.g729800.t1.2.html]

### Cre17.g729800.t1.2

Trivial name: ALB3.2  
SeqCoverage [median]: 32.90  
Mass: 44.74 kDa  
  
 

| Sequence | MissedCleavage | Mass [Da] | wtIntensity | muIntensity | Score |
| --- | --- | --- | --- | --- | --- |
| AASTVSAGAGGSEEGKDNSA | 1 | 1764.78 | 4532.40 | 9462.21 | 307.01 |
| APATAGSSTPIVKPK | 1 | 1423.80 | 23538.69 | 34924.57 | 125.97 |
| APATAGSSTPIVKPKEER | 2 | 1837.99 | 2295.60 | 1007.25 | 48.31 |
| ATVKVNIPEAIK | 1 | 1281.77 | 321.00 | 0.00 | 33.69 |
| IISSQNNQDPSQQQAQAILK | 0 | 2210.13 | 6695.86 | 20872.18 | 182.17 |
| QVESTLSLQALQPR | 0 | 1568.85 | 9265.69 | 11844.82 | 164.81 |
| VATFPLTQK | 0 | 1003.57 | 1284.17 | 5639.76 | 95.81 |
| VNIPEAIK | 0 | 882.52 | 14033.84 | 18229.64 | 143.89 |
| VNIPEAIKAPATAGSSTPIVKPK | 2 | 2288.31 | 4095.65 | 14724.40 | 75.11 |
| YADDPENLQLETAR | 0 | 1633.76 | 19981.30 | 33814.60 | 301.16 |
|  |  |  | 86044.2 | 150519.4 |  |
